# Prognosis of Paradoxical Low-Flow Low-Gradient Aortic Stenosis: A Severe, Non-critical Form, With Surgical Treatment Benefits

**DOI:** 10.3389/fcvm.2022.852954

**Published:** 2022-04-01

**Authors:** Laura Galian-Gay, Roxana Andreina Escalona Silva, Gisela Teixidó-Turà, Guillem Casas, Elena Ferrer-Sistach, Cristina Mitroi, Susana Mingo, Vanessa Monivas, Daniel Saura, Bàrbara Vidal, Livia Trasca, Sergio Moral, Francisco Calvo, Maria Castiñeira Busto, Violeta Sánchez, Ariana Gonzalez, Gabriela Guzman, Marta Noris Mora, MiguelÁngel Arnau Vives, Jesús Peteiro, Alberto Bouzas, Aleksandra Mas-Stachurska, Teresa González-Alujas, Laura Gutiérrez, Rubén Fernandez-Galera, Filipa Valente, Andrea Guala, Aroa Ruiz-Muñoz, Cesar Augusto Sao Avilés, José F. Rodríguez Palomares, Ignacio Ferreira, Artur Evangelista

**Affiliations:** ^1^Department of Cardiology, Hospital Universitari Vall d'Hebron, CIBER-CV, Universitat Autònoma de Barcelona, Barcelona, Spain; ^2^Department of Cardiology, Hospital Universitari Germans Tries i Pujol, Badalona, Spain; ^3^Department of Cardiology, Hospital Puerta de Hierro - Majadahonda, Madrid, Spain; ^4^Department of Cardiology, CIBER-CV, Hospital Clínico Universitario Virgen de la Arrixaca, Murcia, Spain; ^5^Department of Cardiology, Hospital Clínic de Barcelona, Barcelona, Spain; ^6^Department of Cardiology, Hospital Josep Trueta, Girona, Spain; ^7^Department of Cardiology, Hospital Alvaro Cunqueiro, Vigo, Spain; ^8^Department of Cardiology, CIBER-CV, Hospital Universitario 12 de Octubre, Madrid, Spain; ^9^Department of Cardiology, Hospital Ramón y Cajal, Madrid, Spain; ^10^Department of Cardiology, Hospital Universitario La Paz, Madrid, Spain; ^11^Department of Cardiology, Hospital Universitario Son Espases, IdISBa, Mallorca, Spain; ^12^Department of Cardiology, Hospital Universitario y Politécnico La Fe, Valencia, Spain; ^13^Department of Cardiology, CIBER-CV, Complexo Hospitalario Universitario A Coruña, Coruña, Spain; ^14^Department of Cardiology, Hospital del Mar – Parc de Salut Mar, Barcelona, Spain

**Keywords:** aortic stenosis, paradoxical low-flow low-gradient, echocardiography, aortic valve surgery, heart valve disease

## Abstract

**Objectives:**

To determine the risk of mortality and need for aortic valve replacement (AVR) in patients with low-flow low-gradient (LFLG) aortic stenosis (AS).

**Methods:**

A longitudinal multicentre study including consecutive patients with severe AS (aortic valve area [AVA] < 1.0 cm^2^) and normal left ventricular ejection fraction (LVEF). Patients were classified as: high-gradient (HG, mean gradient ≥ 40 mmHg), normal-flow low-gradient (NFLG, mean gradient < 40 mmHg, indexed systolic volume (SVi) > 35 ml/m^2^) and LFLG (mean gradient < 40 mmHg, SVi ≤ 35 ml/m^2^).

**Results:**

Of 1,391 patients, 147 (10.5%) had LFLG, 752 (54.1%) HG, and 492 (35.4%) NFLG. Echocardiographic parameters of the LFLG group showed similar AVA to the HG group but with less severity in the dimensionless index, calcification, and hypertrophy. The HG group required AVR earlier than NFLG (*p* < 0.001) and LFLG (*p* < 0.001), with no differences between LFLG and NFLG groups (*p* = 0.358). Overall mortality was 27.7% (*CI* 95% 25.3–30.1) with no differences among groups (*p* = 0.319). The impact of AVR in terms of overall mortality reduction was observed the most in patients with HG (hazard ratio [*HR*]: 0.17; 95% *CI*: 0.12–0.23; *p* < 0.001), followed by patients with LFLG (*HR*: 0.25; 95% *CI*: 0.13–0.49; *p* < 0.001), and finally patients with NFLG (*HR*: 0.29; 95% *CI*: 0.20–0.44; *p* < 0.001), with a risk reduction of 84, 75, and 71%, respectively.

**Conclusions:**

Paradoxical LFLG AS affects 10.5% of severe AS, and has a lower need for AVR than the HG group and similar to the NFLG group, with no differences in mortality. AVR had a lower impact on LFLG AS compared with HG AS. Therefore, the findings of the present study showed LFLG AS to have an intermediate clinical risk profile between the HG and NFHG groups.

## Introduction

Degenerative aortic stenosis (AS) is the most common valve disease in developed countries and, owing to aging of the population, threatens to become a true epidemic in coming decades (1). Paradoxical low-flow low-gradient (LFLG) AS poses diagnostic challenges and uncertainties regarding the true severity of the disease and appropriate therapeutic decision-making. Initial studies considered LFLG AS to be an entity with worse prognosis than high-gradient (HG) AS and could thus benefit from surgical or percutaneous treatment as or earlier than in HG AS (2–4). However, recent meta-analyses questioned these results, considering that LFLG AS probably behaves in an intermediate manner between moderate and severe AS (5–8). The present study aimed to assess, in a large and contemporary cohort of patients with AS, the natural history and prognosis of LFLG AS in comparison with HG and normal-flow low-gradient (NFLG) AS, as well as the impact of aortic valve replacement (AVR) in each subgroup.

## Materials and Methods

**Figure 1:**
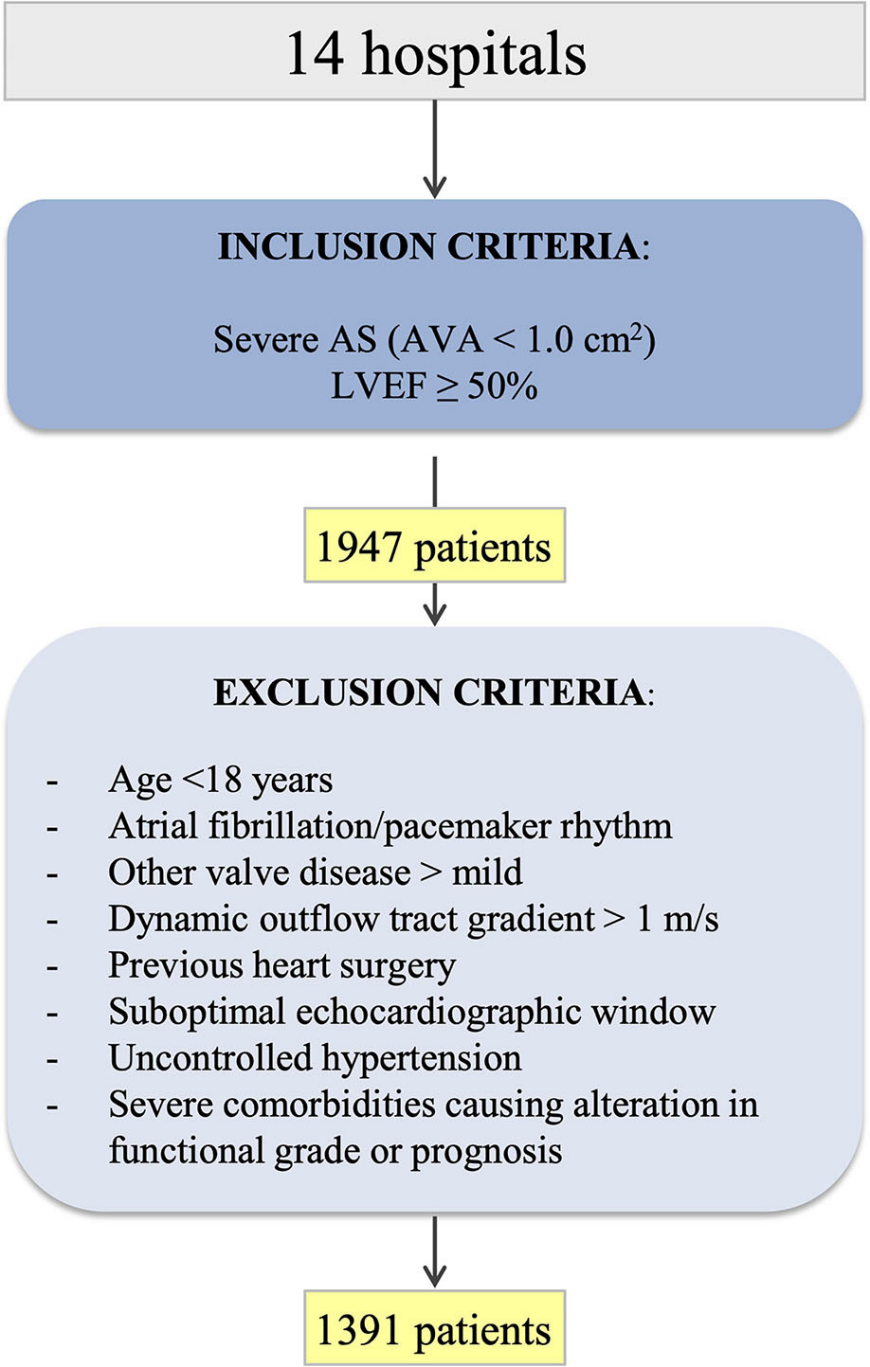
A flowchart of patients participating in the study. AS, *aortic stenosis*; AVA, *aortic valve area*; LVEF, *left ventricular ejection fraction*.

A retrospective longitudinal observational study was conducted of consecutive patients from 14 tertiary hospitals nationwide diagnosed between 2008 and 2016 with severe AS (aortic valve area (AVA) < 1.0 cm^2^) with left ventricular ejection fraction (LVEF) ≥ 50% on the transthoracic ECG (Figure 1). Exclusion criteria were: age < 18 years, atrial fibrillation or pacemaker rhythm, aortic regurgitation more than mild, other valvular heart disease more than mild, left ventricular outflow tract dynamic gradient exceeding a velocity >1 m/s, previous heart surgery, suboptimal echocardiographic window, poor blood pressure control, and comorbidities at baseline that could themselves cause an alteration in functional grade or prognosis (e.g., severe chronic obstructive pulmonary disease [COPD]).

The approval for the study was obtained from the Ethics Committee of the Vall d'Hebron Hospital (PR (AG) 60/2018). The study protocol conformed to the ethical guidelines of the Declaration of Helsinki 1975 as reflected in the Ethics Committee approval.

Baseline demographic and clinical data were collected. The presence of coronary artery disease was defined when a history of acute myocardial infarction, significant ischaemic or the presence of coronary artery disease were documented. The presence of baseline symptoms was considered when the functional status of the New York Heart Association was ≥ II, or syncope or angina was reported in clinical reports. Follow-up clinical data, such as the need for surgery or TAVI, status (alive/deceased), and cause of death (cardiovascular/non-cardiovascular) were also obtained. AVR was indicated at the participating centers according to current guideline indications (9).

Echocardiographic studies were performed in all patients by expert imaging cardiologists at the participating centers. Measurements were obtained following European Association of Cardiovascular Imaging (EACVI) standards and were validated by a senior expert from each center. AVA was calculated through continuity equation. The degree of aortic valve calcification and stenosis was established semi-quantitatively as recommended by current guidelines (10). Patients were classified into 3 groups according to baseline ECG data regarding the gradient and indexed systolic volume (SVi) as recommended by current guidelines (10): high-gradient (HG) if mean gradient ≥ 40 mmHg, normal-flow low-gradient (NFLG) if mean gradient < 40 mmHg and SVi > 35 ml/m^2^, and LFLG if mean gradient < 40 mmHg and SVi ≤ 35 ml/m^2^. Patients were not involved in the design, conducting, reporting, or dissemination plans of our research.

All analyses were made with Stata software version 13.1. Continuous variables were expressed as mean and SD when the normality assumptions were met and as median and interquartile range (IQR) otherwise. Categorical variables were expressed as absolute numbers and percentages. Demographic, echocardiographic, and AVR variables were evaluated among the different groups using ANOVA test, chi-square test, or Fisher's exact test, as appropriate. Kaplan–Meier analysis was used for time-to-event variables (time-mortality and time-AVR) and the log-rank test to compare survival and time-AVR curves among groups. A multivariate Cox regression analysis was used to identify independent variables associated with the response variable (predictors of mortality and AVR), such as variables with statistical significance (*p* < 0.20) in the univariate analysis or with significant clinical relevance.

## Results

### Baseline Clinical and Demographic Characteristics

In this study, 1,391 patients with baseline ECG with AVA < 1 cm^2^ and normal LVEF from 14 tertiary hospitals (Figure 1), mean age 74.5 (10.9) years and 53.6% women were included. In the whole cohort, 752 (54.1%) were classified as HG, 492 (35.4%) as NFLG, and 147 (10.5%) as LFLG. No significant differences were observed among groups regarding age, sex, or cardiovascular risk factors, except for smoking and body weight (Table 1). The mean total follow-up time was 59.0 months (IQR 39.7–82.9 months), with no significant differences among groups.

**Table 1:** Clinical and demographic data according to aortic stenosis (AS) subgroups at baseline.

	**All patients *n =* 1,391**	**HG *n =* 752** **(54.1%)**	**NFLG *n =* 492 (35.4%)**	**LFLG *n =* 147** **(10.5%)**	***p*-value**
Females, *n (%)*	744 (53.6)	393 (52.3)	272 (55.4)	79 (54.5)	0.560
Age, *years* [mean (SD), *median, IQR*]	74.5 (10.9) *77 (70–82)*	74.3 (10.8) *76 (69–82)*	74.4 (11.3) *77 (70–81)*	76.0 (10.4) *78 (73–82)*	0.155
Body surface area, *Kg*/*m^2^*	1.8 (0.2)	1.8 (0.2)	1.7 (0.2)	1.8 (0.2)	0.834
Weight, *Kg*	72.7 (13.3)	73.0 (13.3)	71.1 (12.1)	76.6 (15.6)	<0.001
Height, *cm*	160.3 (9.6)	160.6 (9.9)	159.7 (8.8)	160.3 (10.3)	0.264
Hypertension, *n* (%)	1,120 (80.5)	601 (79.9)	403 (81.9)	116 (78.9)	0.584
Dyslipidaemia, *n* (%)	799 (57.4)	431 (57.3)	284 (57.7)	84 (57.1)	0.987
Diabetes, *n* (%)	456 (32.8)	254 (33.8)	147 (29.9)	55 (37.4)	0.157
Smoking status, *n* (%)	270 (19.4)	169 (22.5)	80 (16.3)	21 (14.3)	0.007
Coronary disease, *n* (%)	320 (23.0)	165 (22.0)	121 (24.6)	34 (23.1)	0.550
COPD, *n* (%)	172 (12.4)	93 (12.4)	65 (13.2)	14 (9.5)	0.513
Baseline symptoms, *n (%)*	777 (55.9)	438 (58.2)	256 (52.0)	83 (56.5)	0.097
Follow-up, *months (IQR)*	59.0 (39.7–82.9)	59.3 (38.4–84.6)	59.4 (43.5–79.8)	55.6 (36.6–76.5)	0.286

*AS, aortic stenosis; COPD, chronic obstructive pulmonary disease; HG, high gradient; IQR, interquartile range; LFLG, low-flow low-gradient; NFLG, normal-flow low-gradient; SD, standard deviation. For continuous variables mean and SD was expressed [mean (SD)]*.

### Baseline Echocardiographic Characteristics

The echocardiographic data of each AS subtype are shown in Table 2. Remarkably, AVA of the LFLG group was similar to AVA of the HG group [0.74 (0.14) vs. 0.73 (0.16) cm^2^; *p* = NS] and significantly lower than that of the NFLG group [0.89 (0.09), *p* < 0.001]. However, the dimensionless index (ratio between LVOT VTI and aortic VTI) value in the LFLG group was intermediate between the HG group [0.25 (0.06) vs. 0.22 (0.05); *p* < 0.001] and the NFLG group [0.25 (0.06) vs. 0.27 (0.04); *p* < 0.001], with differences between the HG and NFLG groups also being significant [0.22 (0.05) vs. 0.27 (0.04); *p* < 0.001]. Left ventricular hypertrophy was significantly lower in patients of the LFLG group compared with the HG group and similar to the NFLG group (Table 2). Severe valve calcification in the LFLG group was lower than in the HG group and showed no significant differences with the NFLG group.

**Table 2:** Echocardiographic data according to the AS subgroup at baseline.

	**All patients** ***n =* 1391**	**HG *n =* 752** **(54.1%)**	**NFLG *n =* 492 (35.4%)**	**LFLG *n =* 147** **(10.5%)**	***p*-value**
Maximum aortic jet velocity, *m/s*	4.4 (0.3)	5.1 (0.7)	3.6 (0.3)	3.5 (0.4)	0.035^a.b^
Mean aortic gradient, *mmHg*	42.0 (14.0)	51.6 (11.5)	31.2 (5.4)	29.2 (6.7)	<0.001^a.b^
LVOT, *mm*	2.04 (0.17)	2.04 (0.17)	2.04 (0.15)	1.97 (0.18)	<0.001^b.c^
LVOT VTI, *cm*	23.3 (4.7)	24 (5.0)	23.6 (3.8)	18.4 (3.7)	<0.001^a.b^
SVi, *ml/m^2^*	43.2 (9.1)	44.7 (9.6)	44.5 (6.2)	30.8 (3.3)	<0.001^b.c^
AVA, *cm^2^*	0.79 (0.16)	0.73 (0.16)	0.89 (0.09)	0.74 (0.14)	<0.001^a.c^
Dimensionless index	0.24 (0.05)	0.22 (0.05)	0.27 (0.04)	0.25 (0.06)	<0.001^a.b.c^
Severe aortic valve calcification, *n* (%)	717 (54.1)	488 (67.7)	186 (38.7)	4 (35.0)	<0.001^a.b^
Bicuspid aortic valve. *n* (%)	156 (11.5)	88 (11.9)	59 (12.3)	9 (6.5)	0.008^b.c^
LVEDD, *mm*	46.0 (6.7)	46.3 (6.8)	45.7 (6.0)	45.2 (7.5)	0.059
LVESD, *mm*	29.0 (6.2)	29.2 (6.2)	28.8 (6.0)	29.2 (6.6)	0.062
IVS, *mm*	13.7 (2.4)	14.2 (2.4)	13.1 (2.4)	13.4 (2.5)	<0.001^a.b^
PW, *mm*	12.3 (2.1)	12.6 (2.1)	11.8 (2.0)	12.1 (2.2)	<0.001^a.b^
LVEF, %	64.8 (7.2)	65.0 (7.3)	64.5 (7.1)	64.6 (7.7)	0.228
LVEF 50–55%, *n* (%)	63 (4.5%)	31 (4.1)	21 (4.3)	11 (7.5)	0.200
LV mass, *g/m^2^*	130.5 (42.5)	139.7 (41.7)	120.9 (41.7)	118.1 (38.8)	<0.001^a.b^
E/e'	14.7 (7.4)	14.6 (8.0)	14.8 (7.3)	14.7 (5.4)	0.881
LA volume, *ml*	72.5 (36.7)	71.6 (26.0)	73.8 (49.6)	74.0 (40.7)	0.510

*AS, aortic stenosis; AVA, aortic valve area; COPD, chronic obstructive pulmonary disease; HG, high gradient; IQR, interquartile range; IVS, interventricular septum; LA, left atrium. LFLG, low-flow low-gradient; LVEDD, left ventricle end diastolic diameter; LVEF, left ventricular ejection fraction; LVESD, left ventricle end systolic diameter; LVOT, left ventricle outflow tract; NFLG, normal-flow low-gradient. PW, posterior wall; SD, standard deviation; SVi, indexed systolic volume; VTI, velocity-time integral. For continuous variables mean and SD was expressed [mean (SD)]. a, significant differences between HG and NFLG; b, significant differences between HG and LFLG; c, significant differences between NFLG and LFLG*.

### AVR Indication According to AS Subgroups at Baseline

**Figure 2:**
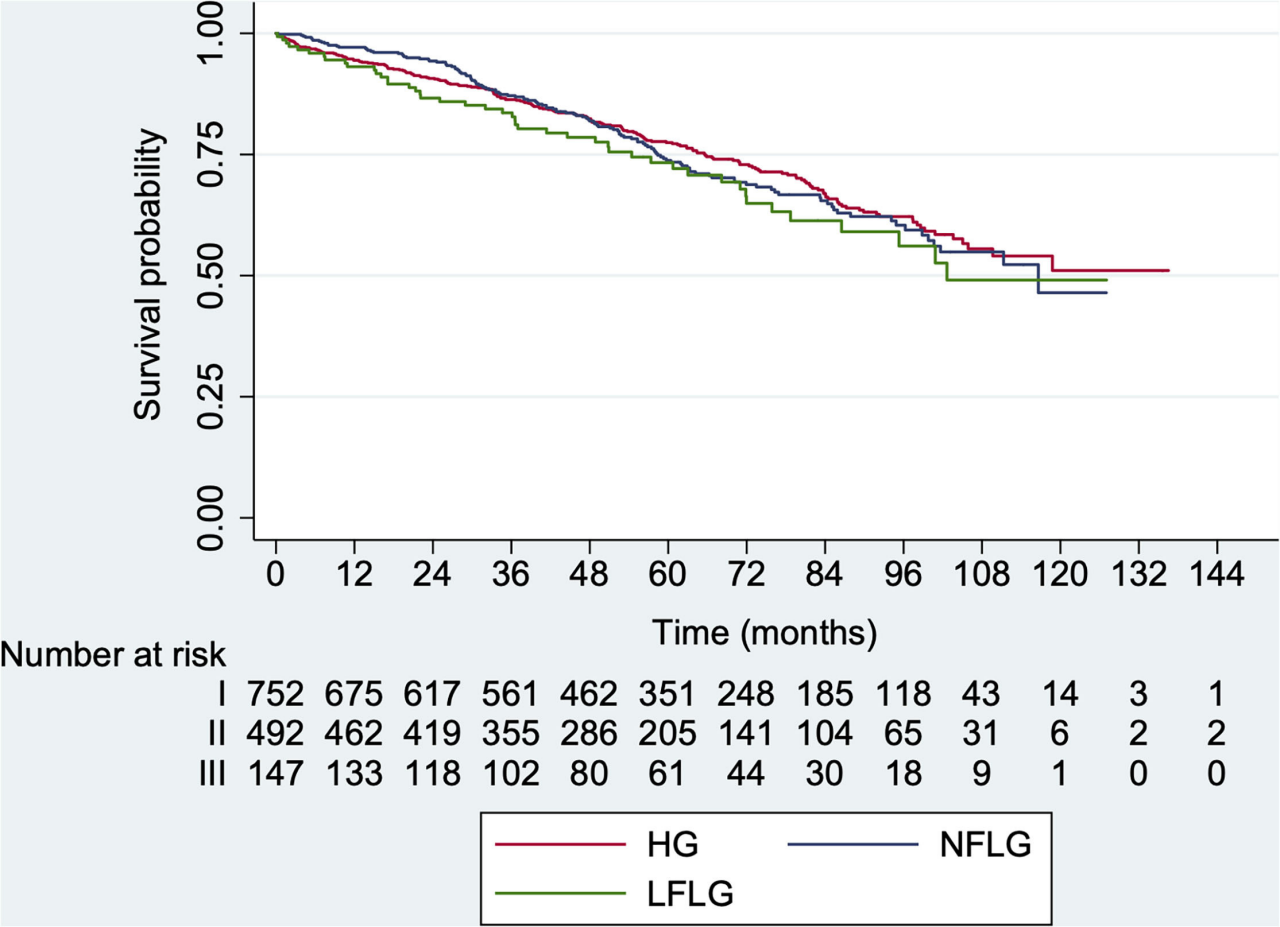
Kaplan–Meier aortic valve replacement (AVR)-free survival curves according to AS subtype at baseline. HG, *high-gradient*; LFLG, *low-flow low-gradient*; NFLG, *normal-flow low-gradient* (log-rank *p* < 0.001).

In total, 1,248 patients had complete data related to AVR (676 (54.2%) with HG, 450 (36.0%) with NFLG and 122 (9.8%) with LFLG). Throughout the follow-up, 857 patients (68.7%, *CI* 95% 66.0–71.2) underwent AVR [685 surgery and 172 Transcater Aortic Valve Implantation (TAVI)]: 529 with HG (78.2%, *CI* 95% 75.0–81.3; median time: 17.7 months, IQR 5.5–43.4 months), 74 with LFLG (60.6%, *CI* 95% 51.4–69.4; median time: 41.0 months, IQR 13.6–78.9 months) and 254 with NFLG (56.4%, *CI* 95% 51.7–61.1; median time: 46.9 months, IQR: 26.0–70.0 months) (Figure 2) with differences among groups in the estimated survival free from AVR that persisted after adjustment for age, smoking, diabetes mellitus, presence of symptoms, and LVEF (*p* < 0.001). In HG AS, AVR was indicated earlier compared with NFLG (log-rank *p* < 0.001) and LFLG AS (log-rank *p* < 0.001). No significant differences were observed between the LFLG and NFLG groups (log-rank *p* = 0.358).

On multivariate analysis, indication of AVR was inversely associated with age (hazard ratio [*HR*] 0.99, *CI* 95%: 0.98–0.99; *p* = 0.002); nevertheless, the presence of symptoms (*HR* 1.82, 95% *CI*: 1.57–2.01; *p* < 0.001) and coronary artery disease (*HR* 1.21, 95% *CI*: 1.03–1.43; *p* = 0.018) were independently related to AVR indication. Regarding echocardiographic parameters, AVA (AVA < 0.8 cm^2^: *HR* 1.25, 95% *CI*: 1.04–1.48; *p* = 0.014), mean gradient (mean gradient ≥ 40 mmHg: *HR* 1.95, 95% *CI*: 1.67–2.29, *p* < 0.001), dimensionless index (dimensionless index ≤ 0.25: *HR* 1, 39, 95% *CI*: 1.18–1.65, *p* < 0.001), and LVEF 50–55% (*HR* 1.52, 95% *CI*: 1.21–1.91, *p* < 0.001) were also independently associated to AVR indication (Supplementary Table 1).

### Mortality According to AS Subgroup at Baseline

**Figure 3:**
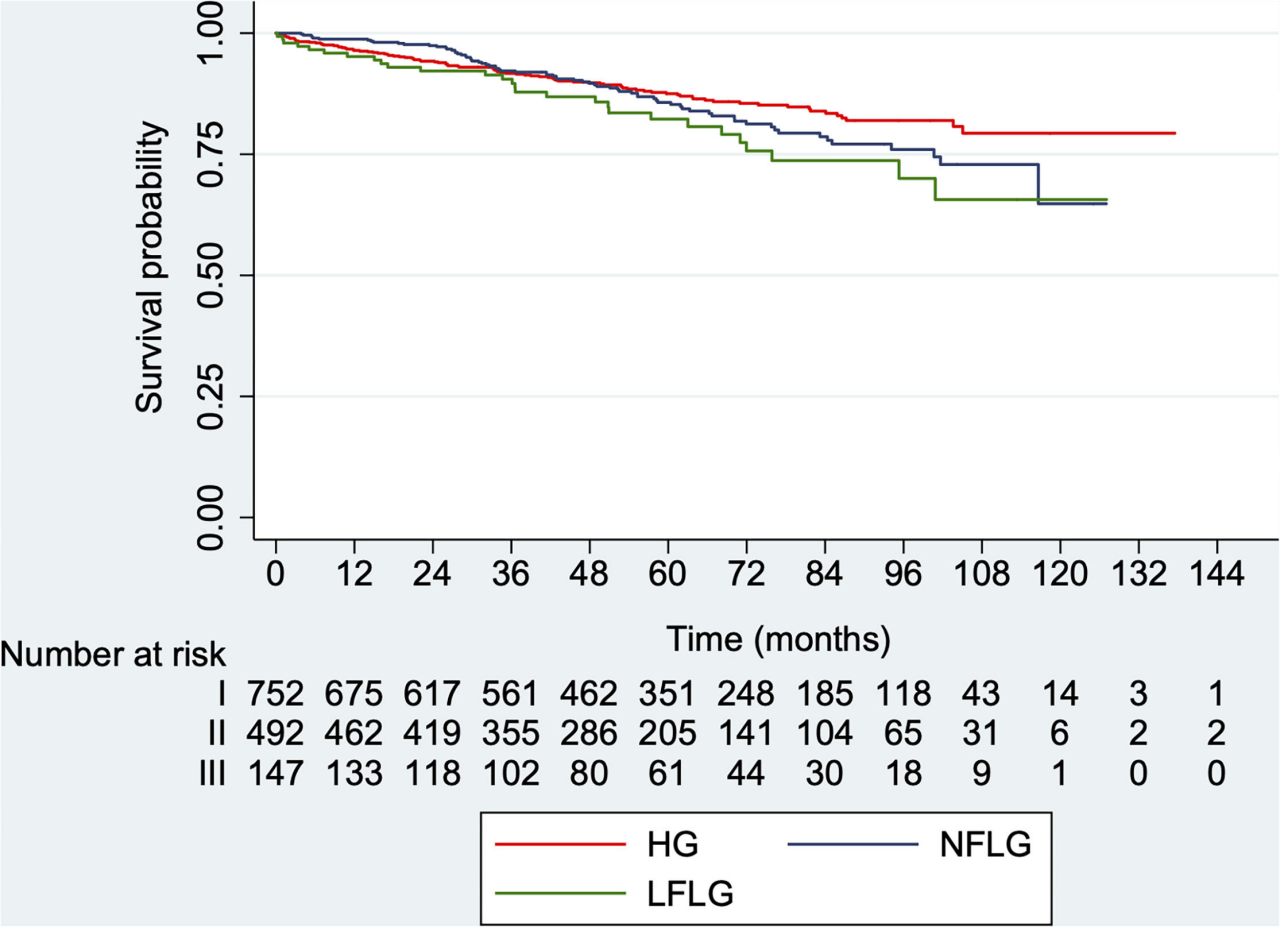
Kaplan–Meier survival curves for overall mortality according to AS subtype at baseline. HG, *high-gradient*; LFLG, *low-flow low-gradient*; NFLG, *normal-flow low-gradient* (log-rank *p* = 0.319).

Overall mortality during follow-up was 27.7% (385 patients, *CI* 95% 25.3–30.1): 46 of the LFLG group (31.3%, *CI* 95% 23.9–39.5; median time: 50.8 months, IQR: 29.6–75.8), 205 of the HG group (27.3%, *CI* 95% 24.1–30.6; median time: 56.1 months, IQR: 33.8–83.7), and 134 of the NFLG group (27.2%, *CI* 95% 23.3–31.4; median time: 53.2 months, IQR: 31.0–76.9). Kaplan–Meier survival curves showed no significant differences among groups (Figure 3, log-rank *p* = 0.319). Early mortality after AVR (<30 days) was 6.5% (25 patients, *CI* 95% 1.2–2.6) with no differences among groups.

**Figure 4:**
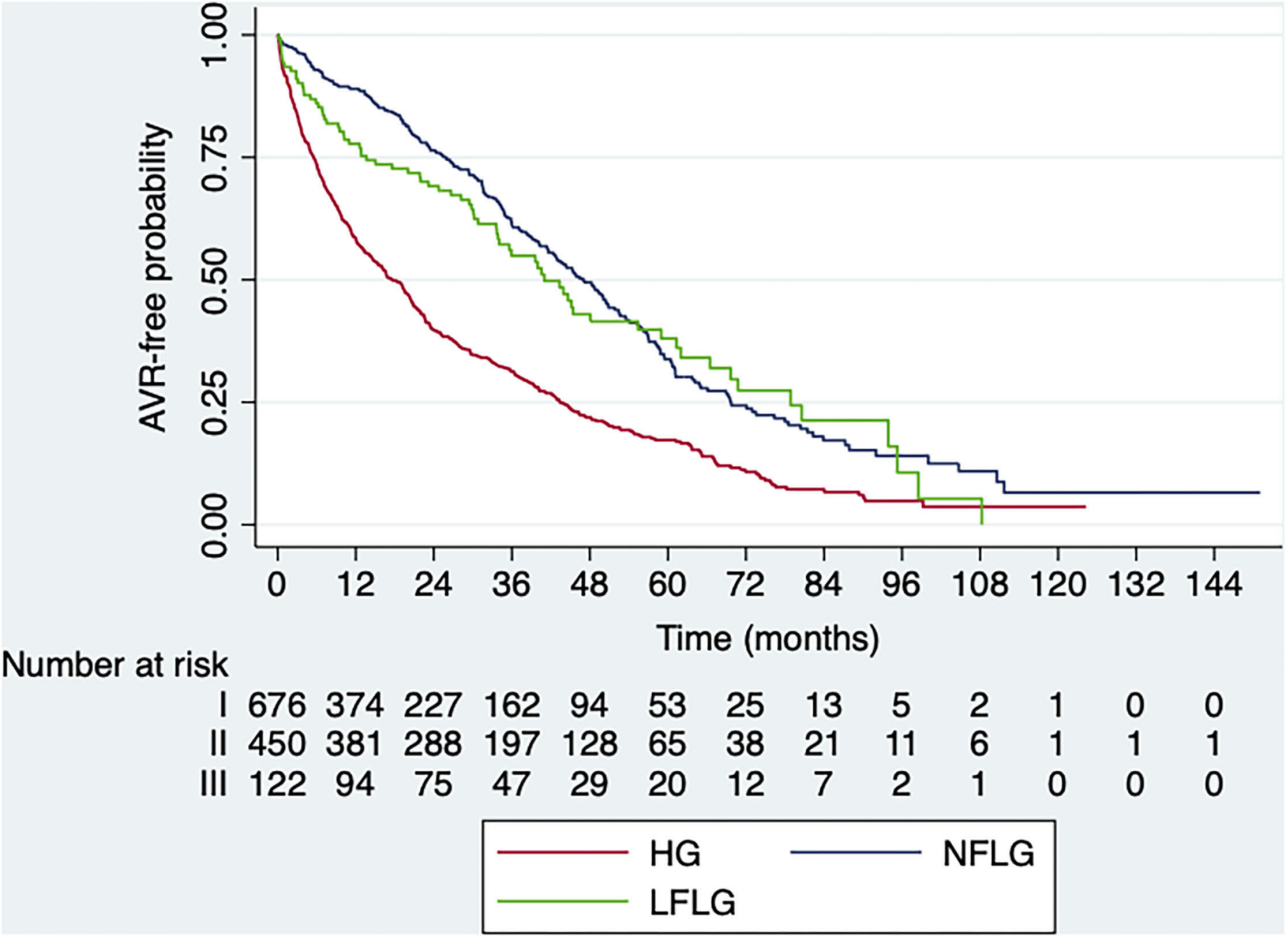
Kaplan–Meier survival curves for cardiovascular mortality according to AS subtype at baseline. HG, high-gradient; LFLG, *low-flow low-gradient*; NFLG, normal-flow low-gradient (log-rank *p* = 0.061).

Death from cardiovascular cause occurred in 195 cases (14%, *CI* 95% 12.2–16.0) representing a 50.6% of overall mortality: 28 patients with LFLG (19%, *CI* 95% 13.0–26.3; median time: 50.9 months, ICR: 32.0–75.9), 95 patients with HG (12.6%, *CI* 10.3–15.2; median time: 56.1 months, ICR: 33.8–83.7), and 72 patients with NFLG (14.6%, *CI* 95% 11.6–18.1; median time: 52.5 months, ICR: 29.0–76.9), with no significant differences among groups (Figure 4, log-rank *p* = 0.061).

In patients undergoing AVR, no significant differences in mortality were observed among groups (log-rank *p* = 0.612) after adjustment for significant clinical variables and AVA. However, in non-operated patients, differences were observed among groups (log-rank *p* = 0.004), with low event-free survival in the HG group compared with the NFLG group (log-rank *p* = 0.001), with no significant differences between the LFLG and HG groups (log-rank *p* = 0.354) or between the LFLG and NFLG groups (log-rank *p* = 0.171).

In the multivariate analysis, age (*HR* 1.06, 95% *CI*: 1.04–1.08; *p* < 0.001), diabetes mellitus (*HR* 1.52; 95% *CI*: 1.23–1.89; *p* < 0.001), smoking (*HR*: 1.77, 95% *CI*: 1.32–2.37; *p* < 0.0001), COPD (*HR* 1.45, 95% *CI*: 1.09–1.92; *p* = 0.010), and the presence of symptoms (*HR* 1.48, 95% *CI*: 1.18–1.85; *p* = 0.001) were clinical variables associated to overall mortality (Supplementary Table 2). Echocardiographic variables independently associated with mortality were mean gradient > 50 mmHg (*HR* 1.56, 95% *CI*: 1.17–2.08; *p* = 0.002) and LVEF 50–55% (*HR* 1.68, 95% *CI*: 1.07–2.63; *p* = 0.023).

### Impact of AVR on Mortality Reduction

**Figure 5:**
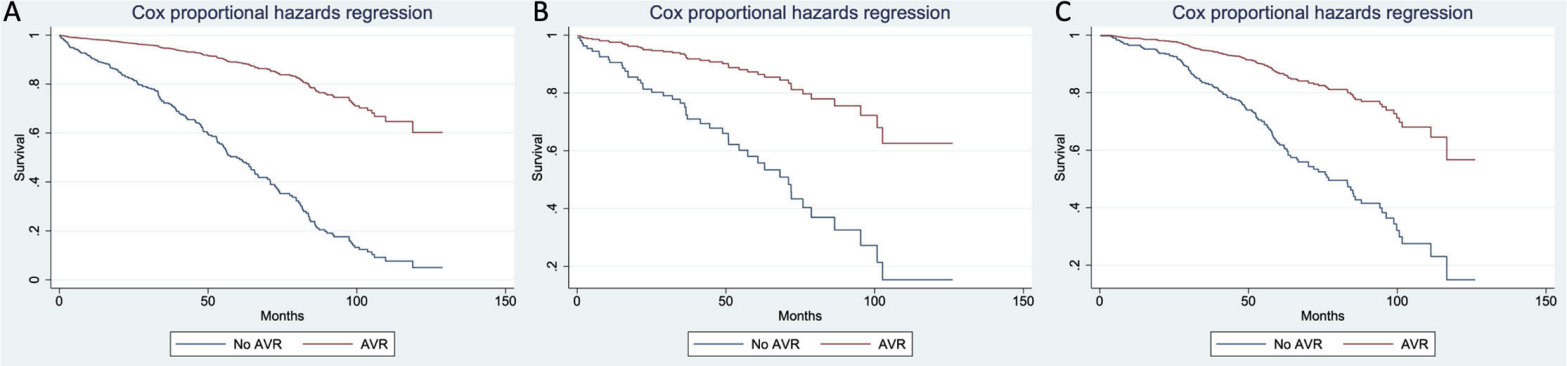
Cox survival curves for overall mortality in the population with severe AS according to AVR and AS subtype [**(A)**: HG; **(B)**: LFLG; **(C)**: NFLG)]. AVR, aortic valve replacement.

Overall mortality was higher in those patients who did not undergo AVR. The impact of AVR on mortality reduction in the whole population with AS was significant (*HR*: 0.22; 95% *CI*: 0.18–0.28; *p* < 0.001) after adjustment for significant clinical variables and AVA. AVR reduced mortality risk by 83% in patients with HG AS (*HR*: 0.17; 95% *CI*: 0.12–0.23; *p* < 0.001), 75% in patients with LFLG (*HR*: 0.25; 95% *CI*: 0.13–0.49; *p* < 0.001), and 71% in patients with NFLG AS (*HR*: 0.29; 95% *CI*: 0.20–0.44; *p* < 0.001; Figure 5).

## Discussion

In this large multicentre retrospective cohort of consecutive patients with AS and normal ejection fraction and sinus rhythm, the prevalence of paradoxical LFLG AS was 10%. Echocardiographic parameters showed a similar AVA in comparison with HG and NFLG groups, but less severity in the dimensionless index, valve calcification, and LV hypertrophy than in HG AS. However, overall/cardiovascular mortality and the impact of AVR on mortality reduction were similar to patients with HG AS.

### Echocardiographic Characterization of LFLG AS

In the present series, patients with LFLG AS had a similar AVA value to the HG group and lower than NFLG group. However, in the remaining echocardiographic parameters, such as left ventricular hypertrophy and degree of valve calcification, LFLG did not significantly differ from NFLG AS. Interestingly, the dimensionless index in LFLG AS laid between HG and NFLG AS. Thus, LFLG AS presented intermediate-severity echocardiographic parameters between HG and NFLG AS. Other series yielded similar results (4). Clavel et al. in a series of 561 patients with AS, found LFLG patients to have significantly lower velocity and transaortic gradients than HG AS while AVA was practically the same (11). Several studies reported that this entity was associated with small ventricles and a high degree of hypertrophy (2); however, the present LFLG group had a significantly lower degree of hypertrophy than patients with HG AS but similar to those with NFLG AS. Other studies using echocardiography or CMR stated that patients with LFLG AS do not have a greater degree of hypertrophy than HG AS, but lower or similar to NFLG AS or moderate AS (4, 5, 12). Few studies analyzed the degree of calcification in LFLG AS by echocardiography or multidetector CT (MDCT), although this is currently one of the more recommended approaches to diagnose severe AS (10, 13). Calcification by MDCT may be highly useful in patients with discordant severity data on echocardiography (13).

### Outcome in LFLG Patients and Protective Effect of AVR

The risk throughout follow-up of undergoing AVR was higher for patients with HG, lower in patients with LFLG AS and comparable to patients with NFLG AS, as previously reported (14). Mortality was 27% and no significant differences were detected between patients with LFLG AS and the other groups. Patients of the three hemodynamic groups who underwent AVR had a similar prognosis, whereas patients who did not undergo surgery had higher mortality in the HG group compared with the NFLG group, with no differences between the LFLG and HG groups. Different series previously reported poorer survival and a higher rate of events in the LFLG population compared with HG AS (3, 11, 15); however, the trend in the more contemporary series has changed the paradigm, suggesting that higher mortality is associated with HG AS, with the behavior of LFLG AS being more similar to moderate AS or intermediate between HG and NFLG AS (8, 16). Recent evidence suggested that moderate forms of AS are not as benign as historically assumed, particularly if left ventricular dysfunction is present (17). A recent study analyzing data from the Australian national echocardiography database showed mortality in patients with moderate AS to be similar to severe AS (18). Another contemporary study reported that patients with NFLG who did not undergo surgery had 6.3 times more overall mortality compared with surgically-treated patients, with surgery being associated with a significant increase in survival (19). Taking the results of this study and previous reported findings into account, there may be sufficient reasons to consider AS severity as moderate-severe when the AVA is between 0.8–1.2 cm^2^ and severe when < 0.8 cm^2^. In moderate-severe patients with AS, other multimodality parameters, such as calcium score by CT, exercise test, or left ventricle overall strain could help to identify a subgroup of patients in whom AVR would be indicated.

Overall, patients with significant AS benefited from intervention on the AV, and despite the HG AS group benefited the most, both LFLG and NFLG AS also obtained significant benefit. Although few studies failed to show this benefit in patients with LFLG AS (5, 20), most series suggested a beneficial effect of valve replacement in the LFLG population (and even in NFLG and moderate AS) compared with conservative management (4, 18, 19). Thus, evidence of the effectiveness of aggressive treatment (surgical or percutaneous) of AS continues to grow and, though data remain discrepant, the trend is toward a more aggressive approach and in a wider range of the disease.

### Mortality Risk Predictors in Patients With Severe AS

In this study, patients with AS who died were older, more symptomatic, had more cardiovascular risk factors and a smaller AVA. However, the multivariate analysis failed to show AVA to be independently associated with mortality which instead was related to age, risk factors (diabetes, smoking, and COPD), symptoms, mean gradient > 50 mmHg, and LVEF from 50–54%. Some studies showed mortality in AS to be associated with maximum velocity, aortic calcification, and LVEF. Valvular heart disease guidelines recommended as class I an AVR indication in systolic dysfunction (LVEF < 50%) regardless of the presence of symptoms (9, 21). However, recent studies reported that patients with LVEF < 55% had poor prognosis (22–24). All these results suggest that the cut-off point of <50% for LVEF could be too low, indicating that left ventricular dysfunction is already present when LVEF is between 50 and 60% and, in fact, recent guidelines recommend that patients with LVEF <60% on serial studies and severe AS should undergo surgery (21). Cardiac magnetic resonance demonstrated the presence of late gadolinium enhancement and increased extracellular volume in AS patients with normal ejection fraction, which have been related to prognosis (25). Other imaging techniques, such as strain by speckle tracking echocardiography can detect patients with subclinical ventricular dysfunction, (26) with overall longitudinal strain values < −15% being associated with worse prognosis (27, 28). Global LV strain values may become one of the markers that will provide additional data to decide whether or not the patient could have a higher risk and deserves surgery. Given that patients with LFLG AS benefit significantly from AVR, they should be followed with caution, with the accuracy in echocardiographic severity evaluation to be maximized to avoid possible errors in SVi quantification. Furthermore, additional information through other imaging techniques (myocardial strain, CMR of calcium score by CT) are likely to be useful to determine whether data support the AS severity and/or suggest incipient ventricular dysfunction, to sustain the choice to treat paradoxical LFLG as HG AS.

## Limitations

The main limitation of this study was its retrospective nature. In the present series, confirmation of the low-flow state was not requested for inclusion. Exclusion of patients with atrial fibrillation was considered in the design of the study since the continuity equation may be less accurate in AVA calculation. Calcification of the aortic valve was analyzed following the semi-quantitative approach recommended by the current guidelines, although the optimal method is cardiac CT. Myocardial strain would have added complementary information; however, owing to the multicentre nature of the study and the years of inclusion established, variability of the values depending on the different vendors used would have resulted in difficult result analysis.

## Conclusions

Low-flow low-gradient AS has intermediate echocardiographic severity parameters and clinical outcomes between NFLG and HG AS, with lower AVR requirements than HG AS, but with overall mortality and benefits of surgery similar to the other two haemodynamic groups. Given that patients classified within this group benefited significantly from AVR, they should be followed with caution as in HG AS. The most appropriate option to adequately manage this subgroup may be to maximize the accuracy of the echocardiographic evaluation, provide additional information through other imaging techniques and determine whether there are data supporting the severity of AS or suggestive of incipient ventricular dysfunction, for them to be treated as HG AS.

## Data Availability Statement

The datasets presented in this article are not readily available because further analyses are still underway for other papers awaiting publication. Requests to access these datasets should be directed to LG-G, lauragaliangay@gmail.com.

## Ethics Statement

The studies involving human participants were reviewed and approved by CEIm - Comitè d'Ètica d'Investigació amb medicaments - VHIR. Informed consent was not available due to the retrospective nature of the study.

## Author Contributions

LG-G, GT-T, GC, and AE designed the study. LG-G, RE, GC, EF-S, CM, SMi, VM, DS, BV, LT, SM, FC, MC, VS, AGo, GG, MM, MA, JP, AB, AM-S, TG-A, LG, RF-G, FV, and AR-M contributed to conduction and reporting of the study. LG-G and AS performed the statistical analysis. AE, AGu, JFP, and IF contributed to manuscript revision. AE and IF are the responsible for the overall content as guarantors. All authors contributed to the article and approved the submitted version.

## Funding

AGu has received funding from the Spanish Ministry of Science, Innovation and Universities (IJC2018- 037349-I).

## Conflict of Interest

The authors declare that the research was conducted in the absence of any commercial or financial relationships that could be construed as a potential conflict of interest.

## Publisher's Note

All claims expressed in this article are solely those of the authors and do not necessarily represent those of their affiliated organizations, or those of the publisher, the editors and the reviewers. Any product that may be evaluated in this article, or claim that may be made by its manufacturer, is not guaranteed or endorsed by the publisher.
