# Supplementary material for: Prognosis of Paradoxical Low-Flow Low-Gradient Aortic Stenosis: A Severe, Non-critical Form, With Surgical Treatment Benefits
Source: Front Cardiovasc Med. 2022 Apr 1;9:852954. doi: 10.3389/fcvm.2022.852954 (PMC9011160; doi:10.3389/fcvm.2022.852954)
Supplement: Supplementary file 1 [file Data_Sheet_1.pdf]

Supplementary material

Table 1. Bivariate and multivariate Cox proportional hazard analysis for AVR

	**BIVARIATE**					**MULTIVARIATE**		
	Yes	No	HR	p-value	CI 95%	HR	p-value	CI 95%
Clinical data								
Age, *years* [mean (SD)]	72.3 (7.6)	78.5 (10.6)	0.99	0.165	0.99-1	0.99	0.002	0-98-0.99
Body surface area, *Kg/m*^{2}	1.8 (0.17)	1.7 (0.2)	1.89	0.001	1.30-2.76	1.08	0.732	0.68-1.72
Males, *n (%)*	452 (50.3)	191 (38.8)	1.26	0.001	1.10-1.44	1.24	0.063	0.99-1.40
Hypertension, *n (%)*	706 (78.5)	414 (84.1)	1.06	0.456	0.90-1.25	-	-	-
Dyslipidaemia, *n (%)*	528 (58.7)	271 (55.1)	1.14	0.058	0.99-1.31	1.13	0.101	0.98-1.30
Diabetes, *n (%)*	295 (32.8)	161 (32.7)	1.06	0.448	0.92-1.22	-	-	-
Smoking status, *n (%)*	193 (21.5)	77 (15.6)	1.19	0.041	1.01-1.40	0.86	0.129	0.72-1.04
Coronary disease, *n (%)*	236 (26.2)	84 (17.1)	1.35	<0.001	1.16-1.57	1.21	0.018	1.03-1.43
COPD, *n (%)*	106 (11.8)	66 (13.4)	1.06	0.563	0.86-1.31	-	-	-
Baseline symptoms, *n (%)*	515 (57.3)	262 (53.2)	1.65	<0.001	1.44-1.89	1.82	<0.001	1.57-2.10
Echocardiographic data								
AVA ≥ 0.8 cm^2 *	459 (51.1)	293 (59.5)	*	*	*	*	*	*
AVA < 0.8 cm^2	440 (48.9)	199 (40.4)	1.75	<0.001	1.53-2.01	1.25	0.014	1.04-1.48
Mean gradient < 40 mmHg *	348 (38.7)	291 (59.1)	*	*	*	*	*	*
Mean gradient ≥ 40 mmHg	550 (61.2)	200 (40.6)	2.15	<0.001	1.87-2.47	1.95	<0.001	1.67-2.29
Dimensionless index > 0.25 *	352 (39.1)	264 (53.7)	*	*	*	-	-	-
Dimensionless index ≤ 0.25	546 (60.7)	227 (46.1)	1.95	<0.001	1.69-2.24	1.39	<0.001	1.18-1.65
SVi > 35 ml/m^2 *	731 (81.3)	401 (81.5)	*	*	*	*	*	*

SVi ≤ 35 ml/m²	167 (18.6)	91 (18.5)	1.16	0.083	0.98-1.39	1.07	0.476	0.88-1.31
LVEF > 55% *	809 (90.1)	443 (90.0)	*	*	*	*	*	*
LVEF 50-55%	89 (9.9)	48 (9.8)	1.53	<0.001	1.22-1.92	1.52	<0.001	1.21-1.91

AVA: *aortic valve area*; AVR: *aortic valve replacement*; COPD: *chronic obstructive pulmonary disease*; LVEF: *left ventricular ejection fraction*; SVi: *indexed systolic volume*

Clinically significant variables and variables with p <0.20 in bivariate analysis were chosen for the multivariate analysis.

* Reference variable for the comparison between groups

Table 2. Bivariate and multivariate Cox proportional hazard analysis for overall mortality

	BIVARIATE					MULTIVARIATE		
	Yes	No	HR	p-value	CI 95%	HR	p-value	CI 95%
Clinical data								
Age, *years* [mean (SD)]	78.5 (7.5)	72.9 (11.6)	1.09	<0.001	1.07-1.11	1.06	<0.001	1.04-1.08
Body surface area, *Kg/m²*	1.7 (0.17)	1.8 (0.18)	0.33	<0.001	0.19-0.58	0.49	0.063	0.24-1.04
Males, *n (%)*	218 (56.6)	526 (52.3)	1.17	0.119	0.96-1.44	1.04	0.773	0.79-1.38
Hypertension, *n (%)*	330 (85.7)	790 (78.5)	1.73	<0.001	1.29-2.3	1.10	0.528	0.82-1.48
Dyslipidaemia, *n (%)*	221 (57.4)	578 (57.5)	1.03	0.758	0.84-1.26	-	-	-
Diabetes, *n (%)*	162 (42.1)	294 (29.2)	1.66	<0.001	1.35-2.03	1.52	<0.001	1.23-1.89
Smoking status, *n (%)*	91 (23.6)	179 (17.8)	1.27	0.048	1.0-1.6	1.77	<0.001	1.32-2.37
Coronary disease, *n (%)*	97 (25.2)	223 (22.2)	1.18	0.160	0.94-1.48	1.19	0.174	0.93-1.52
COPD, *n (%)*	74 (19.2)	98 (9.7)	1.88	<0.001	1.46-2.42	1.45	0.010	1.09-1.92
Baseline symptoms, *n (%)*	255 (66.2)	522 (51.9)	1.73	<0.001	1.4-2.14	1.48	0.001	1.18-1.85
AVR	170 (44.2)	729 (72.5)	0.20	<0.001	0.16-0.24	0.21	<0.001	0.16-0.26
Echocardiographic data								
AVA ≥ 0.8 cm^2 *	196 (50.9)	556 (55.3)	*	*	*	*	*	*
AVA < 0.8 cm^2	189 (49.1)	450 (44.7)	1.24	0.029	1.02-1.52	1.17	0.217	0.91-1.49
Mean gradient< 40 mmHg *	180 (46.7)	459 (45.6)	*	*	*	*	*	*
Mean gradient 40-50 mmHg	112 (29.1)	331 (32.9)	0.82	0.099	0.65-1.04	1.14	0.315	0.88-1.48
Mean gradient> 50 mmHg	93 (24.2)	214 (21.3)	1.07	0.569	0.83-1.38	1.56	0.002	1.17-2.08
Dimensionless index > 0.25 *	171 (44.4)	445 (44.2)	*	*	*	-	-	-
Dimensionless index 0.25-0.20	141 (36.6)	354 (35.2)	1.07	0.552	0.86-1.33	-	-	-

Dimensionless index < 0.20	73 (18.9)	205 (20.4)	1.02	0.910	0.77-1.34	-	-	-
SVi> 35 ml/m² *	305 (79.2)	827 (82.2)	*	*	*	*	*	*
SVi 30-35 ml/m²	54 (14)	115 (11.4)	0.78	0.227	0.52-1.17	1.37	0.055	0.99-1.89
SVi < 30 ml/m²	26 (6.7)	63 (6.3)	1.06	0.810	0.66-1.69	1.36	0.162	0.88-2.12
LVEF > 60% *	251 (65.2)	667 (66.3)	*	*	*	*	*	*
LVEF 56-60%	110 (28.6)	298 (29.6)	1.04	0.744	0.83-1.3	1.05	0.679	0.83-1.32
LVEF 50-55%	22 (5.7)	41 (4.1)	1.5	0.064	0.98-2.33	1.68	0.023	1.07-2.63

AVA: *aortic valve area*; AVR: *aortic valve replacement*; COPD: *chronic obstructive pulmonary disease*; LVEF: *left ventricular ejection fraction*; SVi: *indexed systolic volume*

Clinically significant variables and variables with p <0.20 in bivariate analysis were chosen for the multivariate analysis.

* Reference variable for the comparison between groups
